# Plasma levels of receptor-interacting protein kinase 3 is associated with postoperative acute kidney injury in acute DeBakey type I aortic dissection

**DOI:** 10.1186/s13019-022-01783-0

**Published:** 2022-03-15

**Authors:** Lei Wang, Guodong Zhong, Hao Zhou, Xiaochai Lv, Yi Dong, Xiaoli Wang, Xiaofu Dai, Yanfang Xu, Liangwan Chen

**Affiliations:** 1 Department of Cardiovascular Surgery, Union Hospital, Fujian Medical University, No. 29 Xinquan Road, Gulou District, Fuzhou City, Fujian Province China; 2 Key Laboratory of Cardio-Thoracic Surgery (Fujian Medical University), Fujian Province University, Fuzhou, Fujian Province China; 3 Department of Pathology, The Second People’s Hospital, Fujian University of Traditional Chinese Medicine, Fuzhou, Fujian Province China; 4 Department of Nephrology, First Affiliated Hospital, Fujian Medical University, Fuzhou, Fujian Province China; 5 Fujian Provincial Special Reserve Talents Laboratory, Fuzhou, Fujian Province China

**Keywords:** Acute DeBakey type I aortic dissection, Acute kidney injury, Necroptosis, Receptor-interacting protein kinase 3

## Abstract

**Background:**

Postoperative acute kidney injury (AKI) in acute DeBakey type I aortic dissection (ADIAD) is common but has unclear pathogeneses and limited treatments. Receptor-interacting protein kinase 3 (RIP3), a mediator of necroptosis, is associated with human sepsis-induced and posttraumatic AKI, but its role in human postoperative AKI in ADIAD remains unclear. We assumed that RIP3 levels is associated with postoperative AKI in ADIAD.

**Methods:**

Plasma samples and the clinical data of continuous patients with ADIAD were collected prospectively. The patients were divided into three groups according to AKI stage postoperatively. The plasma RIP3 levels were compared among the groups, and the relationship between RIP3 and serum creatinine (sCr), inflammatory cytokines as well as clinical results were analyzed.

**Results:**

Eighty patients were enrolled. The postoperative and elevated RIP3 levels among the three groups were significantly different (*P* < 0.0001), both with a positive trend across the AKI stage (*P* for trend < 0.001), and they were also independent risk factors for postoperative AKI in ADIAD (*OR* = 1.018 and 1.026, *P* < 0.05). The postoperative RIP3 levels were positively correlated with the aortic crossclamp time (*R* = 0.253, *P* < 0.05); the peak values of sCr, procalcitonin, interleukin-6 and lactate postoperatively; the mechanical ventilation time; and the ICU stay time (*R* = 0.66, 0.369, 0.409, 0.397, 0.474 and 0.435, respectively; all *P* < 0.001). Plasma RIP3 level and sCr were comparable in diagnosing postoperative AKI in ADIAD (*P* = 0.898), and higher postoperative RIP3 level was associated with lower survival rate.

**Conclusion:**

The plasma RIP3 levels are associated with postoperative AKI, inflammatory response and clinical outcomes in ADIAD.

**Supplementary Information:**

The online version contains supplementary material available at 10.1186/s13019-022-01783-0.

## Background

Acute DeBakey type I aortic dissection (ADIAD) is the most critical condition in cardiovascular diseases, with most patients requiring emergency surgery. Cardiac surgery-associated acute kidney injury (AKI) is the second most common cause of AKI, except sepsis [1], and the incidence of postoperative AKI in aortic dissection (AD) is higher than that of other cardiac surgeries [2], as high as 18–67% [3, 4]. Postoperative AKI in AD is also an independent risk factor for postoperative death and major complications [5, 6]. However, its pathogenesis remains unclear, and no reliable drugs or specific treatments are available to prevent or cure AKI, except renal replacement therapy (RRT) [1, 7]. But in fact, the prognosis of RRT after AD repair is not ideal [8]; thus, challenges persist in this field.

Necroptosis is a new form of programmed cell death that is different from necrosis and apoptosis. Receptor-interacting protein 1 (RIP1)-RIP3-mixed lineage kinase domain-like protein (MLKL) is the best-characterized pathway of necroptosis. Stimulus signals promote the formation of necrosome, leading to cell rupture with inflammatory cytokines release. The latter and RIP3 can promote the formation of inflammasomes, leading to a self-amplifying cycle of inflammation and necroptosis [9] called necroinflammation. Recent studies have also found other necroptosis pathways that are independent of RIP1 and MLKL [10], but RIP3 is necessary. Necroptosis is associated with renal ischemia–reperfusion injury and cisplatin-induced AKI [11, 12], and knocking out the RIP3 gene or RIP3 inhibitor can protect mice with renal ischemia–reperfusion injury from severe AKI [11]; thus, necroptosis may be a new therapeutic target for AKI.

The pathogenesis of postoperative AKI in AD is unique. Injury to renal tubular epithelial cells may be caused by comprehensive factors, such as renal malperfusion when the aorta is torn [13], severe secondary inflammation during extracorporeal circulation and surgery [14] and ischemia–reperfusion injury during deep hypothermic circulatory arrest. Fewer necroptosis studies have been reported concerning human AKI. Since necroptotic cells can release RIP3 into the circulation, the plasma RIP3 levels can be detected to reflect necroptosis. Currently, only three human studies have shown that the plasma RIP3 levels are associated with sepsis-induced or posttraumatic AKI [15–17], and several studies have shown that the plasma RIP3 levels are correlated with the severity of sepsis, coronary heart disease or heart failure in humans [18–21]. However, no study has been published on the correlation between postoperative AKI in AD and necroptosis or RIP3. This study primarily aimed to analyze the correlation between the plasma RIP3 levels and postoperative AKI in ADIAD for the first time as far as we know. The secondary aim was to analyze the correlation between the postoperative RIP3 levels and serum creatinine (sCr), inflammatory cytokines and clinical outcomes. We assumed that RIP3 and necroptosis, as well as necroinflammation, play important roles in the occurrence and development of postoperative AKI in ADIAD; thus, plasma RIP3 level may be a new predictor and potential therapeutic targets of postoperative AKI in ADIAD.

## Materials and methods

### Patients

**Fig. 1:**
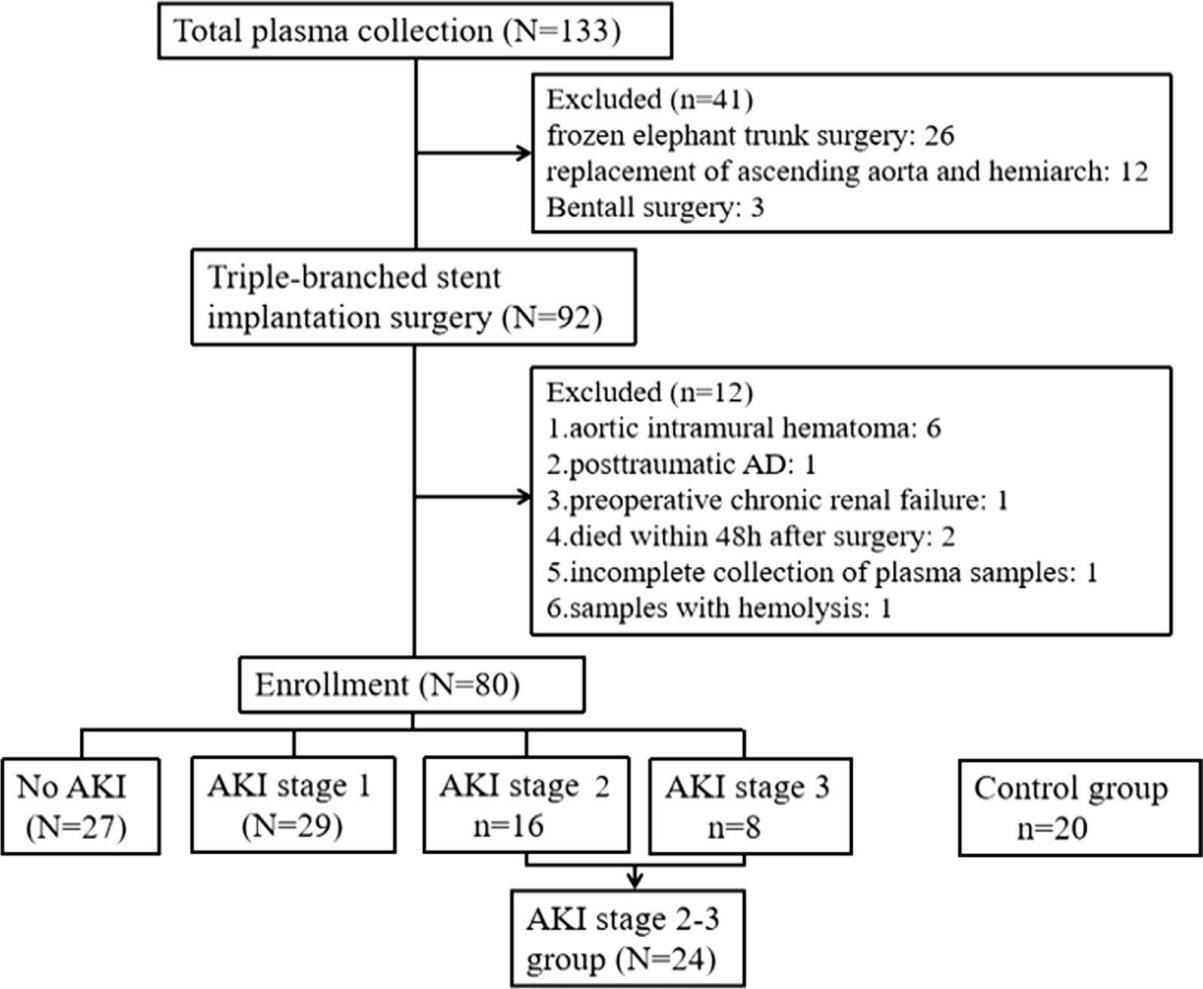
Study flow diagram

This was a prospective observational cohort study. Perioperative plasma samples and clinical data were collected from continuous patients diagnosed with ADIAD in cardiovascular surgery at Union Hospital of Fujian Medical University from August 2020 to January 2021. One hundred and thirty-three patients were enrolled into the cohort, and 80 patients met the inclusion and exclusion criteria. *Inclusion criteria* (1) patients were diagnosed with ADIAD by computed tomography angiography (CTA) of the thoracic and abdominal aorta and echocardiography and received AD surgery under moderate hypothermia circulation arrest (It means the nasopharyngeal temperature is 20–28 °C during circulation arrest) within 5 days of AD onset; and (2) the surgical methods were artificial vascular replacement of the ascending aorta and hemiarch combined with open implantation of a triple-branched stent in the descending aorta pioneered by Professor Liangwan Chen [22]. *Exclusion criteria* (1) aortic intramural hematoma; (2) posttraumatic AD; (3) patients with preoperative chronic renal failure who had received RRT; (4) patients who died or discontinued treatment within 48 h after surgery; and (5) incomplete collection of plasma samples or samples with hemolysis. At the same time, the venous plasma RIP3 levels was detected from 20 age-and-sex matched volunteers as control group from physical examination center at the hospital. The flow chart of the included cases is shown in Fig. 1.

### Definitions

(1) Diagnosis for postoperative AKI: The patients were diagnosed with no-AKI, AKI stage 1, AKI stage 2 and AKI stage 3 according to the Kidney Disease Improving Global Outcomes (KDIGO) criteria [23] within 7 days postoperatively. Because of the relatively small number of patients with AKI stage 3, we combined patients with AKI stage 2 and stage 3 into one group. AKI was defined as a sCr value increased by ≥ 26.5 µmol/L within 48 h or an sCr value increased by 50% within 7 days after surgery compared with the baseline value at admission. (2) Diagnosis for malperfusion of AD: Comprehensive diagnosis was made according to preoperative CTA indicating decreased blood flow or complete artery occlusion, clinical manifestations such as coma, extremity paralysis or abdominal pain, and laboratory test results such as elevated sCr or myocardial enzyme. (3) Preoperative hypotension is defined as mean arterial pressure (MAP) < 75 mmHg, and the severe hypotension is defined as the MAP < 50 mmHg.

### Collection and detection of plasma samples

Five milliliters of radial arterial blood was collected in an EDTA anticoagulant tube preoperatively after anesthesia and approximately 40 h after surgery. The plasma samples were centrifuged immediately (1000 g for 15 min) after collection or refrigerated in a 4 °C refrigerator and centrifuged within 4 h. They were then separated into EP tubes and immediately placed in a − 80 °C refrigerator. The RIP3 levels were measured using commercial enzyme-linked immunosorbent assay kits (CUSABIO, Wuhan, China) within 3 months of sample collection. The tester strictly followed the kit instructions and was blind to the AKI stage of the patients.

### Statistical methods

IBM SPSS 25.0 statistical software and Medcalc software were used for statistical analysis. The measurement data were presented as the mean ± standard deviation (for normally distributed data) or the median with interquartile range (IQR) (for nonnormally distributed data). The categorical data were presented as counts and percentages. One-way analysis of variance (for measurement and normally distributed data), the Kruskal–Wallis test (for measurement and nonnormally distributed data), chi-squared test or Fisher’s exact test (for categorical data) were used to compare the clinical data among the three groups. Bonferroni analysis was used for pairwise comparisons among the three groups, and a linear trend test was performed for the postoperative and elevated RIP3 levels across the three groups. The independent risk factors for postoperative AKI in ADIAD were analyzed by univariate and multivariate logistic regression. Pearson’s or Spearman’s correlation tests were used to analyze the correlation between the plasma RIP3 levels and clinical parameters. Receiver operating characteristic (ROC) curves of sCr and plasma RIP3 levels were compared to predict postoperative AKI. The Youden’s index in the ROC curve was used to determine the optimal cut-off value of plasma RIP3 levels for predicting AKI in ADIAD. The Kaplan–Meier method was used to compare the survival rate among different postoperative RIP3 levels. *P* < 0.05 was considered statistically significant.

## Results

Perioperative clinical data of the patients. The mean age of the normal controls and patients was (53.24 ± 10.23) years versus (54.33 ± 11.80) years (*P* > 0.05); 15 (75.0%) and 58 (72.5%) patients were male, respectively (*P* > 0.05). The median collection time of the postoperative plasma samples was 40 (33–43) hours. The plasma RIP3 levels of all samples were higher than the lowest detectable level (> 15.6 pg/ml), and the RIP3 levels in 2 patients were decreased after surgery, while the others were all elevated. The preoperative plasma RIP3 levels in patients with ADIAD were significantly higher than those in normal controls [(921.5 ± 185.8) pg/ml vs. (761.4 ± 120.6) pg/ml, *P* < 0.01]. The incidence rates of postoperative AKI and continuous RRT (CRRT) were 66.25% and 8.75%, respectively, in 80 patients. The relationship between preoperative plasma RIP3 levels and preoperative clinical manifestations showed that patients with hypotension, higher preoperative sCr and preoperative renal malperfusion had higher preoperative RIP3 levels, but the last of the three had not yet reached a statistical difference (See Additional file 1). The patients were followed up for 60 days after surgery, and three patients died in hospital, and one died out of hospital. The perioperative characteristics and plasma RIP3 levels of patients were shown in Table 1.

**Table 1:** Perioperative characteristics and plasma RIP3 levels of patients (n = 80)

	No AKI (n = 27)	AKI stage 1 (n = 29)	AKI stage 2–3 (n = 24)	*F* or *H* or χ^2^ Value	*P* value
Male sex	20 (74.1)	22 (75.9)	16 (66.7)	0.944	0.624
Age (years)	52.2 ± 13.0	54.3 ± 9.6	56.4 ± 12.7	0.733	0.484
Body mass index (kg/m^2^)	24.8 ± 3.9	25.9 ± 4.0	24.4 ± 4.0	2.000	0.377
*Past medical history*					
Hypertension	16 (59.3)	21 (72.4)	13 (54.1)	3.315	0.191
Cardiovascular surgery	3 (11.1)	0 (0)	2 (8.3)	3.304	0.187
Marfan syndrome	2 (7.4)	0 (0)	1 (4.2)	2.075	0.408
*Preoperative items*					
Left ventricular ejection fraction (%)	64.7 ± 5.2	67.2 ± 4.2	60.8 ± 9.4	7.034	0.039
Pericardial tamponade	2 (7.4)	3 (10.3)	4 (16.7)	1.142	0.613
Hypotension (MAP < 75 mmHg)	4 (14.8)	5 (17.2)	6 (25.0)	1.117	0.591
MAP: 60–75 mmHg	3 (11.1)	4 (13.8)	2 (8.3)	0.402	0.908
MAP ≤ 50 mmHg	1 (3.7)	1 (3.4)	4 (16.7)	3.591	0.175
Duration of hypotension (hours)	4.8 ± 2.1	2.8 ± 0.8	6.8 ± 3.4	3.499	0.063
Serum creatinine (umol/L)	85.5 (60.4–137.2)	79.5 (68.2–114.2)	68.7 (57.2–114.0)	1.825	0.401
Serum creatinine > 135umol/L	7 (25.9)	3 (10.3)	4 (16.7)	2.446	0.341
Procalcitonin (ng/ml)	0.10 (0.06–0.35)	0.05 (0.03–0.15)	0.12 (0.04–0.27)	4.081	0.130
Interleukin-6 (pg/ml)	54.9 (39.3–110.3)	38.3 (31.2–49.3)	55.4 (39.1–89.9)	6.883	0.032
C-reactiveprotein (mg/L)	6.8 (2.9–59.3)	3.4 (2.2–9.0)	10.3 (4.6–23.2)	5.046	0.080
Malperfusion syndrome	7 (25.9)	9 (31.0)	6 (25.0)	0.472	0.813
Cerebral malperfusion	2 (7.4)	4 (13.8)	4 (16.7)	1.136	0.639
Extremity malperfusion	2 (7.4)	3 (10.3)	2 (8.3)	0.323	1.000
Renal malperfusion	1 (3.7)	2 (6.9)	1 (4.2)	0.570	1.000
One sided	0 (0)	2 (6.9)	2 (8.3)	2.253	0.452
Both sided	1 (3.7)	0 (0)	0 (0)	1.827	0.638
A total obstruction	0 (0)	0 (0)	2 (8.3)	3.169	0.087
A stenosis	1 (3.7)	2 (6.9)	0 (0)	1.537	0.771
Mesenteric malperfusion	2 (7.4)	1 (3.4)	2 (8.3)	0.773	0.731
Coronary malperfusion	0 (0)	0 (0)	2 (8.3)	0.472	0.813
*Type of combined surgery*					
Aortic valvuloplasty	17 (63.0)	16 (55.2)	14 (58.3)	0.397	0.820
Bentall	2 (7.4)	3 (10.3)	5 (20.8)	1.809	0.397
Coronary artery bypass grafting	0 (0)	0 (0)	2 (8.3)	2.974	0.096
Mitral valve replacement	0 (0)	0 (0)	1 (4.2)	1.961	0.315
*Intraoperative items*					
Lowest Nasopharyngeal temperature (℃)	23.0 (22.1–23.7)	22.5 (22.0–22.8)	22.9 (22.0–23.1)	5.444	0.066
Extracorporeal circulation time (min)	131.5 (120.5–139.5)	137.0 (117.7–155.0)	153.0 (126.0–165.0)	4.790	0.091
Aortic crossclamp time (min)	51.9 ± 14.4	55.9 ± 16.1	66.0 ± 19.7	4.426	0.015
Moderate hypothermia circulation arrest time (min)	16.0 ± 3.7	15.5 ± 4.7	16.0 ± 3.6	1.026	0.867
Red blood cells transfused volume (U)	4 (4–6)	6 (4–6)	6 (4–8)	11.034	0.004
Plasma transfused volume (ml)	523.08 ± 230.85	603.84 ± 206.36	613.04 ± 294.34	0.981	0.430
*Postoperative items*					
Peak creatinine (umol/L)	103.5 (75.1–141.2)	130.5 (114.6–175.5)	219 (143–474)	23.458	0.000
Peak procalcitonin (ng/ml)	1.72 (0.85–3.78)	1.01 (0.70–3.40)	5.18 (1.91–12.60)	14.870	0.001
Peak interleukin-6 (pg/ml)	163.4 (141.0–273.1)	197.9 (166.4–249.9)	284.2 (270.1–321.7)	18.822	0.000
Peak lactate (mmol/L)	3.10 (2.50–4.30)	3.70 (2.55–4.95)	7.10 (3.68–9.73)	12.095	0.002
Peak C-reactiveprotein (mg/L)	211.09 ± 59.42	229.28 ± 49.75	243.30 ± 59.5	1.943	0.151
Extracorporeal membrane oxygenation	0 (0)	0 (0)	2 (8.3)	2.974	0.096
Mechanical ventilation time (h)	19.0 (14.5–39.7)	21.0 (16.7–34.0)	72.0 (33.2–112.0)	18.172	0.000
ICU stay time (h)	55.0 (39.7–72.0)	57.2 (41.5–89.5)	122.5 (68.5–171.2)	16.297	0.000
In-hospital mortality	0 (0)	0 (0)	3 (12.5)	4.628	0.028
*Plasma RIP3 levels (pg/ml)*					
Preoperative RIP3 levels	905.4 ± 183.9	877.7 ± 134.0	987.7 ± 223.7	2.820	0.138
Postoperative RIP3 levels	1007.4 ± 195.8	1101.3 ± 141.4	1394.5 ± 160.6	34.331	0.000
Elevated RIP3 levels	101.9 ± 66.7	223.6 ± 89.5	406.8 ± 159.1	44.947	0.000

Data are presented as mean ± standard deviation or median (interquartile range) or n (%)

*MAP* mean arterial pressure, *RIP3* receptor-interacting protein-3

**Fig. 2:**
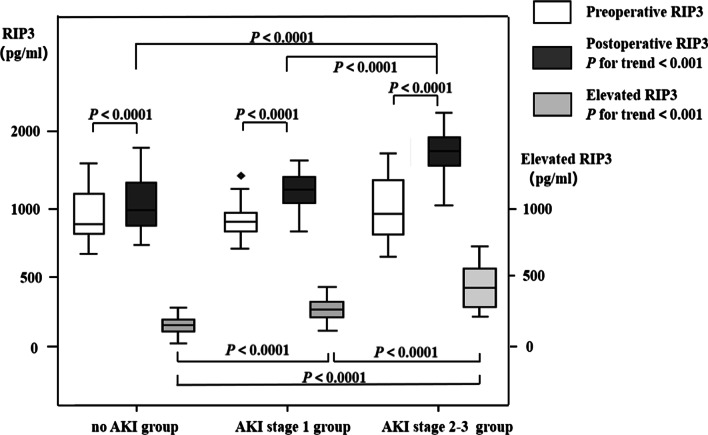
Comparisons of preoperative, postoperative and elevated RIP3 levels among the three groups. This is a double axis chart with boxplot showing preoperative and postoperative RIP3 levels (left vertical axis) and elevated RIP3 levels (right vertical axis). RIP3 levels are presented as median value (black line in the box), interquartile range (box), and maximum and minimum values (upper and lower black line). RIP3 = receptor-interacting protein-3

Comparison of the plasma RIP3 levels among the three groups. The plasma RIP3 levels were significantly higher in the three groups after surgery than before surgery (*P* < 0.001). No differences were found in the preoperative plasma RIP3 levels among the three groups, but there were significant differences in postoperative and elevated RIP3 levels (*P* < 0.0001) with positive linear trends across the AKI stage (*P* for trend < 0.001) among the three groups. Pairwise comparisons of postoperative RIP3 levels among the three groups showed significant differences between the no-AKI and AKI stage 1 groups and between the no-AKI and AKI stage 2–3 groups (*P* < 0.0001). And pairwise comparisons of elevated RIP3 levels all showed significant differences between any two of the three groups (*P* < 0.0001) (Table 1, Fig. 2).

Risk factors for the postoperative AKI in ADIAD. The perioperative factors that might affect the occurrence of postoperative AKI in ADIAD were included in univariate and multivariate logistic regression, and the independent risk factors were the postoperative RIP3 level (*OR* = 1.018; 95% *CI*: 1.009–1.023; *P* < 0.05), the elevated RIP3 level (*OR* = 1.026; 95% *CI*: 1.012–1.040; *P* < 0.05), and the aortic crossclamp time (ACCT) (*OR* = 1.067; 95% *CI*: 1.003–1.134; *P* < 0.05) (Table 2).

**Table 2:** Univariate and multivariate logistic regression analysis of postoperative acute kidney injury in Debakey type I aortic dissection

	Univariate		Multivariate	
	*OR (95%CI)*	*P* value	*OR (95%CI)*	*P* value
Age	–	0.292	–	–
Body mass index	–	0.671	–	–
Extracorporeal circulation time	–	0.146	–	–
Aortic crossclamp time	1.006 (1.003–1.009)	0.044	1.067 (1.003–1.134)	0.039
Red blood cell transfusion volume intraoperatively	1.598 (1.122–2.276)	0.009	–	0.256
Plasma transfusion volume intraoperatively	–	0.169	–	–
Peak lactate values within 7 days postoperatively	1.040 (1.325–1.687)	0.023	–	0.651
Peak interleukin-6 values within 7 days postoperatively	–	0.075	–	0.119
Peak C-reactiveprotein values within 7 days postoperatively	–	0.084	–	0.087
Postoperative RIP3 levels about 40 h postoperatively	1.006 (1.003–1.009)	0.000	1.018 (1.009–1.023)	0.012
Elevated RIP3 levels	1.023 (1.012–1.034)	0.000	1.026 (1.012–1.040)	0.002

*CI* confidence interval, *OR* odds ratio, *RIP3* receptor-interacting protein-3

**Fig. 3:**
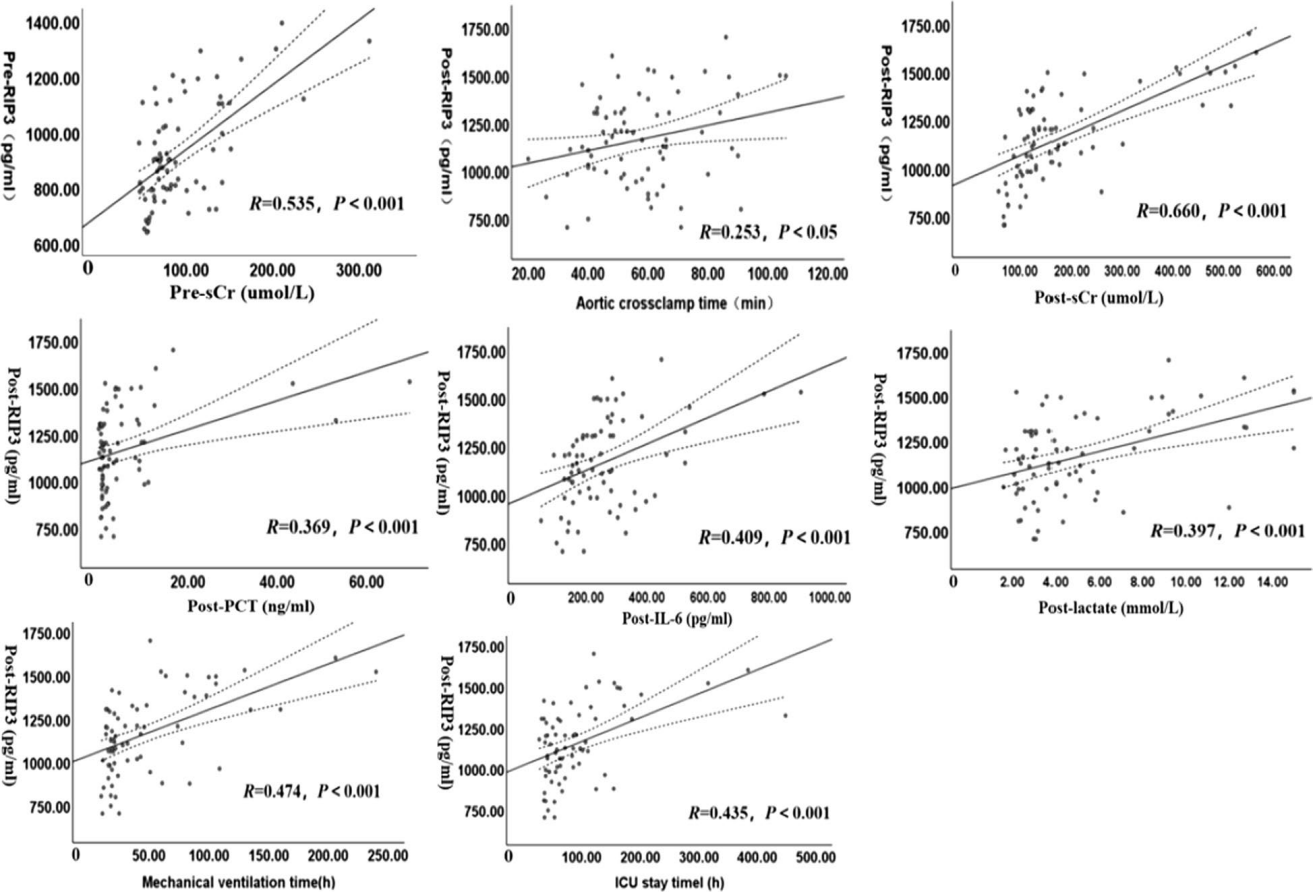
Correlations between RIP3 levels with sCr, aortic crossclamp time, inflammatory cytokines and clinical outcomes. Linear regression of RIP3 levels association with sCr, aortic crossclamp time, PCT, IL-6, lactate, the mechanical ventilation time and ICU stay time, shown as a black line with dashed line area representing 95% pointwise CI. Pre-RIP3 = preoperative receptor-interacting protein-3; Pre-sCr = preoperative serum creatinine; Post-RIP3 = postoperative receptor-interacting protein-3; Post-sCr = postoperative serum creatinine; Post-PCT = postoperative procalcitonin; Post-IL-6 = postoperative interleukin-6; Post-lactate = postoperative lactate

Correlation of the RIP3 levels with clinical parameters. The preoperative RIP3 levels were positively correlated with preoperative sCr levels (*R* = 0.535; *P* < 0.001); The postoperative RIP3 levels were positively correlated with ACCT (*R* = 0.253; *P* < 0.05); the peak values of sCr, procalcitonin (PCT), interleukin-6 (IL-6) and lactate within 7 days postoperatively; the mechanical ventilation time; and the ICU stay time (*R* = 0.66, 0.369, 0.409, 0.397, 0.474 and 0.435, respectively; all *P* < 0.001) (Fig. 3).

**Fig. 4:**
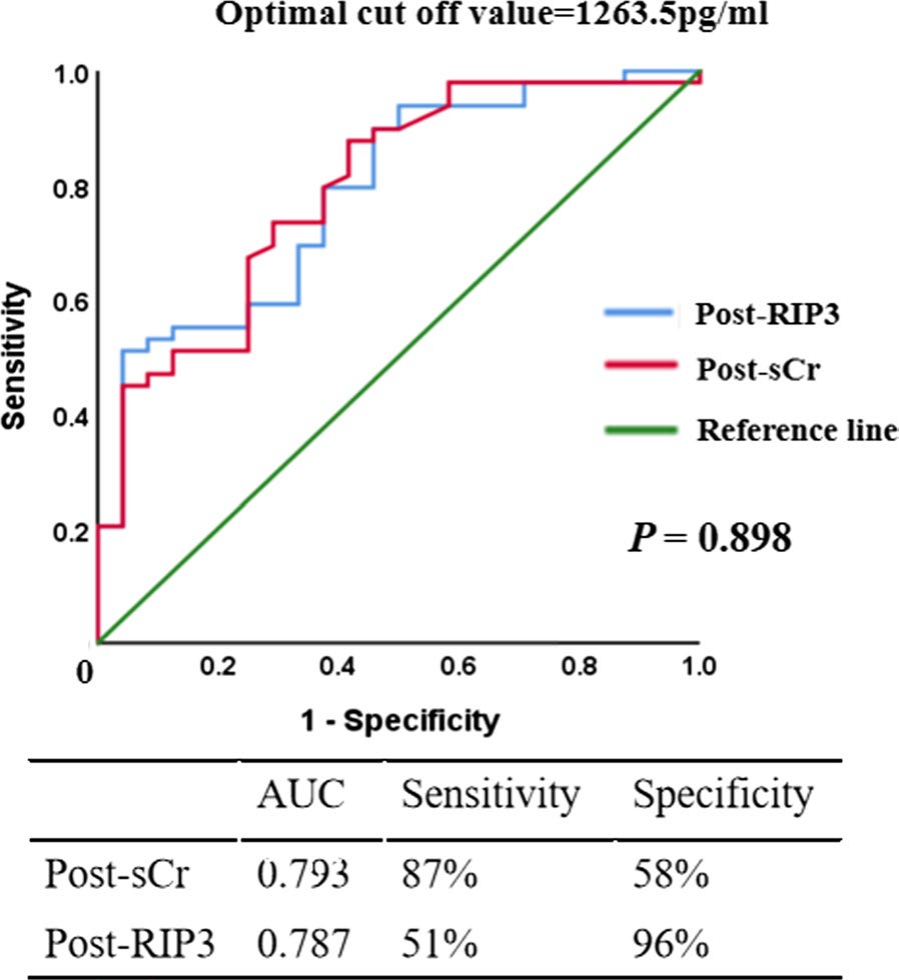
Comparision of the ROC curves. Comparision of the ROC curves between postoperative RIP3 levels and the peak value of postoperative serum creatinine for the diagnosis of postoperative AKI in ADIAD. AUC = The area under the ROC curve, Post-sCr = the peak value of postoperative serum creatinine, Post-RIP3 = postoperative receptor-interacting protein-3

**Fig. 5:**
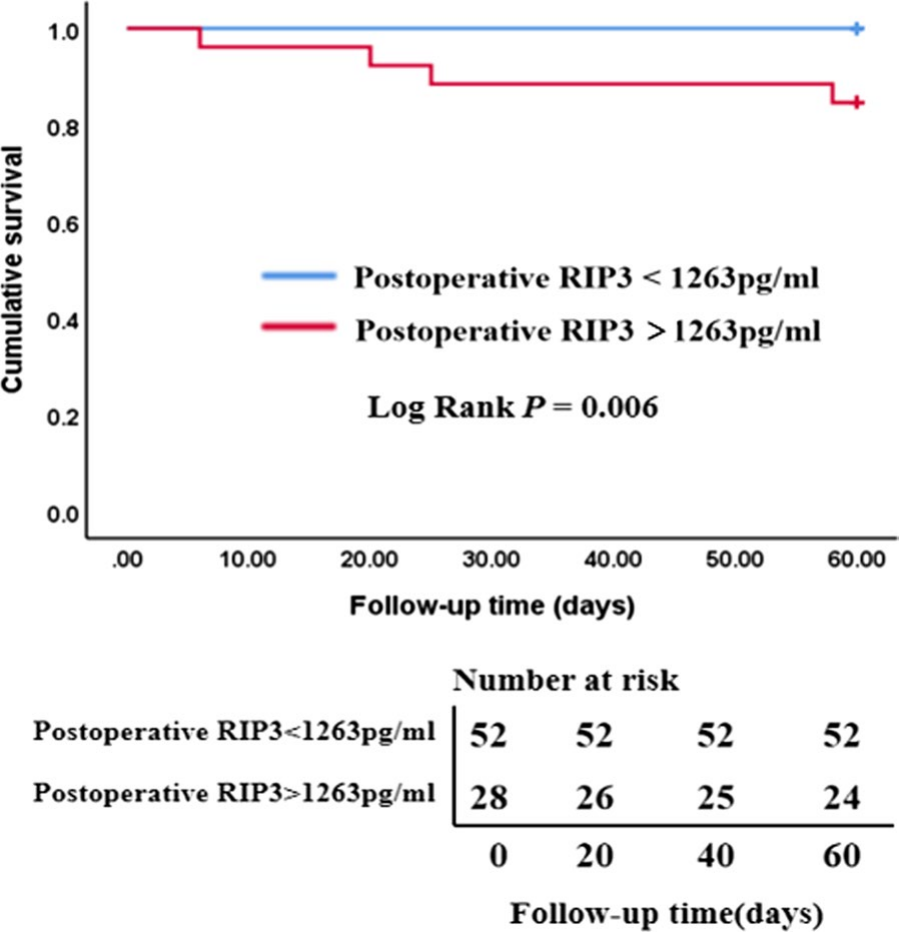
Survival analysis of different postoperative RIP3 levels. In the group of RIP3 levels > 1263 pg/ml, 3 patients died in the hospital, and 1 patient died at 58 days postoperatively. And in the group of RIP3 levels < 1263 pg/ml, no patients died within 60 days postoperatively

Since there was a significant positive correlation between plasma RIP3 and sCr levels, the ROC curves were further made and indicating that the plasma RIP3 was comparable to sCr in diagnosing postoperative AKI in ADIAD (*P* = 0.898) (Fig. 4). According to the ROC curve, the optimal cut-off value of postoperative plasma RIP3 level in predicting postoperative AKI in ADIAD was 1263 pg/ml. Accordingly, the patients were divided into a group with RIP3 levels > 1263 pg/ml and a group with RIP3 levels < 1263 pg/ml. The survival rate at 60 days after surgery was lower in the group with higher postoperative RIP3 levels (LogRank *P* < 0.05) (Fig. 5)

## Discussion

In this cohort study, only ADIAD patients undergoing triple-branched stent implantation surgery were included, and patients with aortic intramural hematoma with relatively lower inflammatory response were excluded, which may interfere with RIP3 levels associated with inflammation. Our study showed that the preoperative plasma RIP3 levels were associated with preoperative hypotension, severe preoperative hypotension and higher preoperative sCr levels. This might be related to prerenal injury due to preoperative hypotension. However, there was no difference in the incidence, the severity or the duration of preoperative hypotension, as well as malperfusion syndrome among the three groups. This might be due to the surgery repair improves renal perfusion or some perioperative factors, for example, the ACCT, were more closely associated with postoperative AKI. The preoperative plasma RIP3 levels in renal malperfusion group was higher, but with no statistical significance, which might be due to only one sided renal malperfusion or both sided renal malperfusion with vascular stenoses and not total obstructions, so the kidney function might be compensated. In total, the patients were relatively homogeneous in the three groups.

The plasma RIP3 level was higher than control groups, and the postoperative RIP3 levels were all higher than preoperative RIP3 levels in the different AKI stage groups. This could be explained by a secondary inflammatory response. There was no difference in preoperative plasma RIP3 levels among the three groups, but the postoperative and elevated RIP3 levels were significantly different among the three groups and showed positive linear trends across the AKI stage. Additionally, the preoperative and postoperative RIP3 levels were positively correlated with sCr, and the comparison of ROC curves of this two biomakers further showed that the plasma RIP3 levels was similar to sCr in diagnosing postoperative AKI in ADIAD. All these might indicate that the surgery and extracorporeal circulation promoted the necroptosis in kidney, and the postoperative plasma RIP3 levels might originate from the injured kidney. Hence the postoperative plasma RIP3 levels might show as a new biomaker in postoperative AKI in ADIAD.

Our study also indicated that the postoperative and elevated plasma RIP3 levels and ACCT were independent risk factors for postoperative AKI in ADIAD, and the postoperative RIP3 levels were significantly positively correlated with the ACCT. This was consistent with the research review [1] indicating that ACCT is an independent risk factor for postoperative AKI in ADIAD. The longer the ACCT was, the longer the times of surgery, extracorporeal circulation and myocardial ischemia were. These effects could lead to more severe systemic inflammation, a necroptosis process that affects the recovery of postoperative cardiac function, malperfusion of visceral organs and more severe postoperative AKI. So necroptosis may participate in the occurrence and development of postoperative AKI in ADIAD during surgery and extracorporeal circulation. Additionally, our study also showed that the preoperative hypotension were associated with higher plasma RIP3 levels. This could also be explained by the fact that the cardiac function could affect renal perfusion.

In addition, our study showed that the plasma RIP3 levels were significantly positively correlated with postoperative inflammatory cytokines such as PCT and IL-6, a finding that was consistent with a study showing a positive correlation between the plasma RIP3 and PCT levels in sepsis patients [19]. Both PCT and IL-6 are acute reactive proteins with increased expression in the body under stress, and they are also increased in patients with AD [24, 25]. The correlation between the plasma RIP3 levels and inflammatory cytokines also reflected the correlation between necroptosis and necroinflammation. A self-amplifying positive feedback cycle exists between necroptosis and necroinflammation, and RIP3 has a proinflammatory effect independent of the function of necroptosis [26, 27]. Inhibition of RIP3 can inhibit necroptosis and necroinflammation [28]. The significant positive correlation between the plasma RIP3 levels and PCT and IL-6 showed that all three could serve as human hematology markers of necroptosis and necroinflammation [29]. Additionally, necroinflammation caused by necroptosis may further aggravate the occurrence and development of postoperative AKI in ADIAD, as well as prolong the postoperative mechanical ventilation time and ICU stay time, affecting the survival rate of patients. This finding was consistent with the results that the plasma RIP3 levels are correlated with the critical condition and prognosis in patients with coronary heart disease and heart failure [20, 21]. Therefore, inhibiting necroptosis and controlling inflammation could be therapeutic targets for postoperative AKI in ADIAD, and the effect of controlling inflammation might be better and faster than that of inhibiting necroptosis pathways [29].

Notably, some plasma samples were collected when the patients had undergone CRRT or extracorporeal membrane oxygenation (ECMO), during which the patient’s blood was in direct contact with the surface of abiotic materials, significantly activating the inflammatory reaction of the body [30, 31]. However, CRRT and ECMO can lead to blood dilution and pipeline adsorption of cytokines, and CRRT can filter some of the inflammatory cytokines [32]; thus, these factors may exert certain effects on the levels of RIP3 and inflammatory cytokines. However, in our study, the levels of inflammatory cytokines in patients who had undergone CRRT or ECMO were still very high, possibly because the plasma collection time was relatively early after surgery, and the severity of the inflammatory reaction in patients was significantly stronger than that of filtrating inflammatory cytokines, blood dilution and pipeline adsorption by CRRT.

The strengths of this study: The prospective cohort study and the included relatively homogeneous patients reduce the confounding bias. The limitations of this study: (1) The sample size was small. (2) Postoperative RIP3 was detected at only one time point without dynamic detection. (3) The urine RIP3 levels did not be detected. (4) Although there was a correlation between RIP3 levels and postoperative AKI stage in our study, no gold standard exists currently to detect necroptosis in humans. The elevated RIP3 levels cannot prove causally that necroptosis directly leads to the occurrence and development of postoperative AKI in ADIAD; further animal studies are warranted.

## Conclusions

This study first analyzed the relationship between the plasma RIP3 levels and human postoperative AKI in ADIAD. The postoperative and elevated RIP3 levels were correlated with the severity of postoperative AKI, and postoperative RIP3 levels were correlated with inflammatory cytokines and patient clinical outcomes. These results suggest that necroptosis and necroinflammation may be involved in the occurrence and development of postoperative AKI in ADIAD, and they might be a new biomaker and potential therapeutic targets for postoperative AKI in ADIAD.

## Supplementary Information

**Additional file 1.** The relationship between preoperative plasma RIP3 level and patients' clinical manifestations.

## Data Availability

The datasets during and/or analysed during the current study available from the corresponding author on reasonable request.

## Abbreviations

RIP3: Receptor-interacting protein kinase 3
AKI: Acute kidney injury
ADIAD: Acute DeBakey type I aortic dissection
sCr: Serum creatinine
AD: Aortic dissection
RRT: Renal replacement therapy
RIP1: Receptor-interacting protein 1
MLKL: Mixed lineage kinase domain-like protein
CTA: Computed tomography angiography
KDIGO: Kidney disease improving global outcomes
MAP: Mean arterial pressure
IQR: Interquartile range
ROC: Receiver operating characteristic
CRRT: Continuous RRT
ACCT: Aortic crossclamp time
PCT: Procalcitonin
IL-6: Interleukin-6
ECMO: Extracorporeal membrane oxygenation
