# Supplementary material for: Plasma levels of receptor-interacting protein kinase 3 is associated with postoperative acute kidney injury in acute DeBakey type I aortic dissection
Source: J Cardiothorac Surg. 2022 Mar 15;17:35. doi: 10.1186/s13019-022-01783-0 (PMC8922876; doi:10.1186/s13019-022-01783-0)
Supplement: Supplementary file 1 — Additional file 1. The relationship between preoperative plasma RIP3 level and patients' clinical manifestations. [file 13019_2022_1783_MOESM1_ESM.docx]

**Additional file 1**

Table 1 The relationship between preoperative plasma RIP3 level and patients' clinical manifestations (n=80)

|  | Yes | No | *P* Value |
| --- | --- | --- | --- |
| [Pericardial](D:/Program%20Files%20(x86)/Youdao/Dict/8.9.6.0/resultui/html/index.html#/javascript:;) [tamponade](D:/Program%20Files%20(x86)/Youdao/Dict/8.9.6.0/resultui/html/index.html#/javascript:;) | 980.6 ± 153.2 | 918.9 ± 187.6 | 0.578 |
| Hypotension (MAP＜75mmHg) | 1008.3 ± 52.1 | 899.1 ± 23.2 | 0.034 |
| [severe](javascript:;) [hypotension](javascript:;) (MAP＜50 mmHg) | 1202.4 ± 64.0 | 896.3 ± 20.4 | 0.001 |
| Creatinine >135umol/L | 1107.2 ± 190.35 | 877.4 ± 156.3 | 0.000 |
| Malperfusion syndrome | 926.5 ± 186.7 | 919.3 ± 187.2 | 0.879 |
| Cerebral malperfusion | 952.8 ± 212.9 | 917.6 ± 183.7 | 0.617 |
| Extremity malperfusion | 824.1 ± 105.3 | 931.8 ± 190.0 | 0.146 |
| Renal malperfusion | 1066.2 ± 201.7 | 916.0 ± 179.6 | 0.098 |
| Mesenteric malperfusion | 969.9 ± 148.8 | 917.9 ± 188.7 | 0.550 |
| [Coronary](D:/Program%20Files%20(x86)/Youdao/Dict/8.9.6.0/resultui/html/index.html#/javascript:;) malperfusion | 819.3 ± 199.4 | 924.3 ± 186.1 | 0.334 |

RIP3= receptor-interacting protein-3.
